# Physical Therapy for Neurological Conditions in Geriatric Populations

**DOI:** 10.3389/fpubh.2017.00333

**Published:** 2017-12-07

**Authors:** Eli Carmeli

**Affiliations:** ^1^Department of Physical Therapy, University of Haifa, Haifa, Israel

**Keywords:** physical therapy, aging, neurology, motor learning, brain

## Abstract

With more of the world’s population surviving longer, individuals often face age-related neurology disorders and decline of function that can affect lifestyle and well-being. Despite neurophysiological changes affecting the brain function and structure, the aged brain, in some degree, can learn and relearn due to neuroplasticity. Recent advances in rehabilitation techniques have produced better functional outcomes in age-related neurological conditions. Physical therapy (PT) of the elderly individual focuses in particular on sensory–motor impairments, postural control coordination, and prevention of sarcopenia. Geriatric PT has a significant influence on quality of life, independent living, and life expectancy. However, in many developed and developing countries, the profession of PT is underfunded and understaffed. This article provides a brief overview on (a) age-related disease of central nervous system and (b) the principles, approaches, and doctrines of motor skill learning and point out the most common treatment models that PTs use for neurological patients.

## Introduction

With increased age, individuals often face age-related neurological decline as well as disorders that can affect activities of daily living (ADL), general function such as gait and balance, and well-being (1). Hence, preserving brain, muscle, and neuromuscular function is critical to health and quality of life.

Neurorehabilitation research has progressed substantially over recent decades. Lauenroth et al. (2) have indicated that neuroplasticity, or the ability of the brain to restructure synaptic connections, specifically in reaction to learning or experience or following injury is a process that occurs throughout the lifespan, even among the aged (3).

The amount of research focusing on motor learning structural and/or functional brain alterations in old people is increasing (4). The knowledge of changes in brain state in neuropathological conditions becomes particularly interesting when the motor learning ability is translated into functional ability (5).

Regarding the involvement of physical therapy (PT) in neurological patients, there are several treatment methods that available for the neurorehabilitation (6). A commonly applied treatment is neurodevelopmental treatment (NDT) (7). PT for the elderly neurologically involved patient with sensory–motor impairments, postural control (i.e., balance), and coordination, and it does so through the knowledge of motor learning and motor control (8). The PT is part of an interdisciplinary team targeted to prevent functional decline, restore function, and ADL, prevent secondary complications and comorbidities, allow compensating to offset and adapt to residual disabilities, and to maintain of function over the long term. The prevention of falls, frailty, fatigue, and sarcopenia could improve the patient’s health and life span (9, 10). PT for neurological patients also has a role in immediate or acute care, when there is a requirement to provide hospital-based short-term intensive PT aimed at the recovery of musculoskeletal and neurological function, limbs positioning, and handling due to hypertonic or spastic muscles (11).

Many factors are associated with the lack of compliance with a PT regimen in the elderly or with the availability of the service. This could be attributed due to internal and external impediments such as insufficient time, malnutrition, lack of motivation, no pleasure while exercising, fear of falling, and lack of social support, no space to exercise, limited finances, no transportation, and so forth. Such reasons can impede achieving the maximum benefits from PT (12). Cognitive impairment, such as dementia and delirium, and psychological impairment such as depression and anxiety can additionally affect the patient’s neurorehabilitation goals and outcomes (13, 14).

The major aim of this mini review is to describe the role of PT in neurorehabilitation for elderly people and to introduce the main rehabilitation approaches and techniques of doing so.

## PT in Developed and Developing Countries

In many developed and developing countries, the profession of PT is significantly underfunded, underestimated, and understaffed (15). These results in either low quality of physiotherapy and unavailable PT services, long waiting periods and in many cases patients seek therapy in (evidence-based treatment options, which frequently worsen the individual’s overall health status). Unfortunately, unsubstantiated treatments and unavailable medicine, practitioners, and health services are situations all too common in many Third World countries (mostly in Africa and some in mid-Asia) where those in poverty cannot afford to establish modern, evidence-based medical services, and where adequate training for physicians and allied health-care providers is not up to the highest standards. Moreover, in these health-and-welfare-deprived countries, national policy and regulations, roles, standard of care, absence of internship accommodations, and medical regulations are not adequately enforced, allowing unsubstantiated practices even flourish and replace universally accepted interventions (16).

The first step in overcoming the shortage of health services in general and skilled PTs in particular is to open academic PT programs that train students according the Commission on Accreditation in Physical Therapy Education (17) guidelines (e.g., 4 years program for pursuing Bachelor PT that contain approximately 3,000 academic hours and 1,000 h of supervised clinical internship). Simultaneously, policy makers must create national board exam along with official documentation to define job description, code of ethics, duties, responsibilities, and scope of practice for PT. Second step is to develop research fields relate to PT, such as gait and balance analysis lab, muscle strength/power objective evaluation tools, electron diagnostic instruments to measure certain qualities such as quantitative electroencephalography and electromyography both in academia and in clinical setting.

## Aging Brain

Not infrequently seen in the aging brain are momentary (i.e., transient ischemic attack) (18), permanent impairment, functional disability, and personality changes (19), which can vary greatly in severity and progression. Aging brains may oftentimes be associated with deficiencies in various regions of the nervous system responsible for vestibular function and motor control (e.g., reduced reaction time, impaired coordinative movements), speech and language function (e.g., anomias), thinking (e.g., confusion, disorientation), sensory perception, learning, mental fatigue, attention, judgment, problem solving (e.g., agnosias, apraxias), ADL and instrumental ADL (e.g., dressing, eating, personal hygiene, shopping, house work, transportation) sleeping, mood (e.g., depression and melancholy), behavioral changes (e.g., stress, anxiety, confusion or delirium, fear, loneliness/isolation) (20), and disorganized behavior and doing unusual things (e.g., shouting, undressing in public).

## Neuroplasticity

Despite physiological and structural changes affecting the brain tissue, the aged brain, to some degree, can learn and relearn due to dynamic events known as “neuroplasticity” (21). Neuroplasticity can occur by producing certain proteins such as brain-derived neurotrophic factor, by evolving new connections between synapses and forming new pathways in the central nervous system. Although neuroplasticity emerges more often right after birth and during the first years of life (22), our brain’s ability to learn new skills, to relearn old skills, and to adapt activities continues frequently also as we aged; however, the ability, quality, and rate of learning and relearning are expected to diminish and proceed at a slower pace. Neuroplasticity is likely due to two major neurophysiological processes: neurogenesis and synaptogenesis (23).

## Age-Related Neurology Disorders

Neurodegenerative conditions are a general term for a range of conditions, which primarily affect neurons in the central nervous system, both at the brain and spinal cord level. Neurodegenerative diseases such as dementia, Alzheimer’s, Parkinson, and amyotrophic lateral sclerosis (Lou Gehrig’s disease) are incurable and debilitating conditions that result in progressive degeneration and or even death of nerve cells (24).

Other neurological or pathological conditions affecting the brain such as CVA (i.e., stroke), seizure, or any condition composed of the intracranial components of the cerebral cortex: white matter, thalamus, amygdala, hypothalamus, brain stem, and cerebellum, is associated with movement impairments, visual–motor learning (25) communication difficulties, loss of cognitive abilities such as memory and decision making, psychological changes demonstrates strong association between anxiety and depression and fear of falls (26), and decline in function and social participation.

## PT for Neurorehabilitation and Neuro-Maintenance by Didactic Means

In general, the PT uses the International Classification of Functioning, Disability, and Health model in a problem solving approach to assess activity, function, and participation. In this way, the PT can identify and prioritize relevant needs, concerns, and expectations as a basis for establishing achievable outcomes with patients and caregivers (27, 28). Therefore, in addition to specific neurotreatment, PT includes activities to maintain general fitness, muscle strength and length, aerobic capacity, good posture and postural control, and education of the patients and caregivers about the disease and how to reinforce PT strategies for preventing falls and inactivity, and ways in which to prevent secondary complications such as contractures, and leg ulcers and swelling. Furthermore, one can educate formal and unpaid/family caregivers about safe techniques for lifting and transfer and how to assist with bed mobility and environmental restructuring is indispensable (29). In addition, as necessary, PT prescribes appropriate wheelchairs, chairs, bed mattresses, walking aids, orthopedic shoes, and other assistive technologies and devices.

## Motor Learning

Motor learning in the elderly is not simply applied, and for successful neurorehabilitation the aged individual requires the capacity to learn acquire new information and recall that information, to practice and train with many repetitions [i.e., with many combinations (when the order of the exercises does not matter) and with permutation (when the order does matter) (30, 31)]. The accomplishment of motor skills involves a process of motor control and motor learning. Motor control theories and principles provide an integrated framework from different disciplines such as psychology, neurology, biomechanics, occupational therapy, and physical education (32).

Older adults must frequently accommodate to the gradual deterioration of their sensory–motor systems, emotional and cognitive functions that occur associated with aging, adjust how they perform multitasks and how they manage their health (33). Individuals in the fourth age with associated neurological conditions may need to relearn previously acquired motor skills such as bathing, eating, dressing up, or keeping hygiene with limited and distorted quality of resources available and accessible to them.

Motor skill learning involves many principles, approaches, and doctrines (34). Formal and informal learning is a process of change and dependent on intrinsic plasticity and neuronal dynamics (35) rather than on a collection of accurate and practical knowledge, where a patient is gaining new knowledge, functions, tasks, or skills. Progresses eventually require patience and persistence and tend to follow learning curves. For an individual’s survival, motor development, maintenance of skill and learning are essential and crucial, yet they are typically learned and performed done circumstantially. Motor skills both learned and relearned are not all acquired at once, but build upon and are shaped by what already has been experienced and is known and by the need and drive to truly achieve them. As the brain learns it undergoing electrical, chemical, and structural changes (e.g., signal transducing adapter proteins, G-proteins and ion channels, intra-membrane receptors) that finally, produce a relative permanent change representing long-term procedural/implicit memory.

## Biofeedback in PT

The question that arises in motor learning is what is the best way to learn sensory–motor skills? One of the most helpful techniques is the use of biofeedback (36–38). A biofeedback system recommend external (i.e., augmented information that is an on demanded learning technique which provided by an external source) and intrinsic feedback is response-produced training that is (a) interactive, (b) safe, and (c) allows the individual motivation to discover and relearn motor skills and thus to regain cognitive capacities. External feedback is often categorized as “knowledge of performance” (KP) also known as kinematic feedback, and “knowledge of results” (KR) augmented information (39).

Knowledge of performance refers to information provided to a patient during the activity/task/movement, and it includes information about suitability, accuracy, efficiency, quickness, and velocity. KR is augmented information provided to a patient, verbally and non-verbally after the activity/task/movement was concluded. KR focuses at the success level of the task, so eventually it provides a quantity score (%, points, etc.). Typically, KR feedback can be vocal (“well done,” “great job”) auditory (applause) or visual (such as smiley, or thumb up for good performance and thumb down for poor performance). Carmeli (40) had noted that for biofeedback to be most successful and beneficial for geriatric populations, an individual must include several functional factors such as motivation, challenge point framework, guidance, and should be proven by evidence-based practice. Feedback can improve neurorehabilitation if attention, task-related memory, and “reaction time” are practiced.

Motor learning requires many combinations and permutations. For new synapses and pathways to be formed and for functional connections to be created and developed, neurons must be aroused. Specific ways of administering feedback can activate neurogenesis. Training the brain by repeated and varied practices facilitates positive results. Lisa Muratori and Ben Sidaway described the five necessary categories for efficient motor learning practice including (1) blocked or obstructed practice is when the patient perform a single/identical skill over; (2) by contrast, the patient works on a number of different tasks in combination with each other; (3) distributed practice is when the patient receives more rest time than practice time; (4) massed practice is when the patient does more practice than rest time; and (5) contextual interference—a series of skills are practiced in a random sequence (41, 42).

## Task Analysis and Task-Specific Training

Task-specific or task-oriented practice is an approach to rehabilitation that focuses on performance of functional tasks that are meaningful to the individual (43). In many neurological conditions, specifically in Parkinson’s disease, knowledge of the biomechanics of movement can be used to make certain that the most efficient strategy is trained. It is equally important for PT to occur within the context of functional tasks such as walking (with variations in movement speed, direction, and distance), stairs climbing, standing up from a sitting position, sitting down, turning around, obstacle negotiation, to pick up products from the shelf in the supermarket and place them in a cart, to hang clothes on a clothesline, insert and remove products from the refrigerator, picking up objects off the floor or counter, reaching for a glass, grasping bottle, drinking from cups of different sizes and shapes and manipulating objects with different sizes, shapes, textures, and weights.

Functional training is effective in enhancing transfer and retention and very helpful when there is a high degree of similarity between the trained task and new variations of the task. Moreover, task-specific training also means that PT takes place not in a formal environment such as a PT clinic or laboratory but in the natural environment where the individual’s functional movements are most difficult to perform yet most important for maintaining effective ADL skills (e.g., person’s home/bedroom/kitchen, street, sidewalk/curbs). But, if such environment is not available, PT can provide environmental modifications such as creating ramps, rails, stairs, and different walking surfaces (grass field, asphalt, sand track, gravel path).

There is currently no evidence-based PT to cure neurodegeneration diseases. However, a PT can provide specific means for relieving the symptoms and incapacities of a pathological condition, provide a means of preventing musculoskeletal side effects, and help to improve patients’ quality of life. For example, psychomotor exercises such as Tai Chi can improve cognitive function in older people at risk of cognitive decline (44), and gait training for Parkinson can increase postural stability and reducing muscle rigidity (45).

## The NDT/Bobath (NDT)

Neurodevelopmental treatment is a holistic clinical practice that emphasizes individualized therapeutic handling based on movement analysis for rehabilitation. NDT is based on knowledge of human movement patterns, including atypical patterns, and in-depth knowledge in analyzing postural control, righting reactions, motor learning, associated movements, and activation of key points of control (46, 47). Movement facilitation is accomplished by handling techniques, weight-bearing exercises to guide patients through initiation and completion of intended task. Thus, patients learn how to control postures and movements and then progress to more difficult ones. However, two associates, Prof. Mindy Levin and Ms. Elia Panturin, clearly stated, “…*that a major barrier to the evaluation of the therapeutic effectiveness of the Bobath concept is lack of a unified framework for both experimental identification and treatment of neurological motor deficits*” (48).

## Constraint-Induced Movement Therapy (CIMT)

In this rehabilitation model, initially designed for adults post-stroke, the unaffected arm is restrained, requiring the individual to use the affected side to complete numerous repetitions of various tasks that challenge the system (49, 50). Study outcomes that investigated the effect of CIMT have demonstrated that intense structured practice leads to improvements in function, quality of movement, reaction time, and even changes in the neurosubstrates of the brain, which correspond to improved movement capabilities.

## Proprioceptive Neuromuscular Facilitation (PNF)

Proprioceptive neuromuscular facilitation is a common stretching and strengthening practice with broad applications in treating patients with neurological and musculoskeletal conditions mainly for increasing muscle elasticity and endurance, and improve active, passive range of motions, to increase joint stability, to enhance neuromuscular coordination and control in the athletic and clinical setting (51, 52). When performed in addition to prescribed exercise, PNF may also increase muscular performance. Two PNF techniques are mostly used include the contract–relax method and the contract–relax–antagonist–contract method. For more information about PNF techniques, please see Ref. (53).

## In Summary

Physical neurorehabilitation can enhance brain and neuromuscular adaptation in the fourth age. PT for neurological patients is a comprehensive process that intends to teach, guide, and promote brain plasticity, thus reducing the threats for any functional and cognitive variations (54, 55).

Although there is strong support that a structural PT program for neuropatients could actually affect brain plasticity by assisting neurogenerative, neuroadaptive, and neuroprotective processes. Neurorehabilitation may be implemented in the framework recommended by the International Classification of Function, Health and Diseases. The final goal of gerontology-based PT neurorehabilitation is to improve quality of life for those in the fourth age and, together with global health education programming, to allow individuals the most independence possible and social participation.

## Author Contributions

The author confirms being the sole contributor of this work and approved it for publication.

## Conflict of Interest Statement

The author declares that the research was conducted in the absence of any commercial or financial relationships that could be construed as a potential conflict of interest.

## References

[1] Warsch JR Wright CB. The aging mind: vascular health in normal cognitive aging. J Am Geriatr Soc (2010) 58(Suppl 2):S319–24. 10.1111/j.1532-5415.2010.02983.x 21029061

[2] Lauenroth A Ioannidis AE Teichmann B Influence of combined physical and cognitive training on cognition: a systematic review. BMC Geriatr (2016) 16:141 10.1186/s12877-016-0315-1 27431673 PMC4950255

[3] Ballesteros S Kraft E Santana S Tziraki C. Maintaining older brain functionality: a targeted review. Neurosci Biobehav Rev (2015) 55:453–77. 10.1016/j.neubiorev.2015.06.008 26054789

[4] Takeuchi N Oouchida Y Izumi S. Motor control and neural plasticity through interhemispheric interactions. Neural Plast (2012) 2012:823285. 10.1155/2012/823285 23326685 PMC3541646

[5] Maes C Gooijers J Orban de Xivry JJ Swinnen SP Boisgontier MP. Two hands, one brain, and aging. Neurosci Biobehav Rev (2017) 75:234–56. 10.1016/j.neubiorev.2017.01.052 28188888

[6] Best JR Rosano C Aizenstein HJ Tian Q Boudreau RM Ayonayon HN Long-term changes in time spent walking and subsequent cognitive and structural brain changes in older adults. Neurobiol Aging (2017) 57:153–61. 10.1016/j.neurobiolaging.2017.05.023 28648916 PMC5564417

[7] Oh DS Choi JD. The effect of motor imagery training for trunk movements on trunk muscle control and proprioception in stroke patients. J Phys Ther Sci (2017) 29(7):1224–8. 10.1589/jpts.29.1224 28744053 PMC5509597

[8] Shimamura N Katagai T Kakuta K Matsuda N Katayama K Fujiwara N Rehabilitation and the neural network after stroke. Transl Stroke Res (2017) 8(6):507–14. 10.1007/s12975-017-0550-6 28681346

[9] Carmeli E Frailty and primary sarcopenia. Adv Exp Med Biol (2017) 1020:53–68. 10.1007/5584_2017_18 28382607

[10] Kalron A Allali G Gait and cognitive impairments in multiple sclerosis: the specific contribution of falls and fear of falling. J Neural Transm (Vienna) (2017) 124(11):1407–16. 10.1007/s00702-017-1765-0 28735370

[11] Langhorne P Baylan S. Early supported discharge services for people with acute stroke. Cochrane Database Syst Rev (2017) 7:CD000443. 10.1002/14651858.CD000443.pub4 28703869 PMC6483472

[12] Nicholson S Sniehotta FF van Wijck F Greig CA Johnston M McMurdo ME A systematic review of perceived barriers and motivators to physical activity after stroke. Int J Stroke (2013) 8(5):357–64. 10.1111/j.1747-4949.2012.00880.x 22974010

[13] Severinsen KD Tufton A Hannan E Schwind JS Schmucker D Cutler A. Evaluating outcomes from an integrated health service for older patients. Ochsner J (2015) 15(4):423–8. 26730227 PMC4679304

[14] Haworth J Young C Thornton E. The effects of an ‘exercise and education’ programme on exercise self-efficacy and levels of independent activity in adults with acquired neurological pathologies: an exploratory, randomized study. Clin Rehabil (2009) 23(4):371–83. 10.1177/0269215508101728 19293292

[15] Pechak C Thompson M Disability and Rehabilitation in Developing Countries. Global Health Education Consortium. (2007). Available from: https://www.cugh.org/sites/default/files/105_Disability_and_Rehabilitation_in_Developing_Countries_FINAL_0.pdf

[16] Shields M Quilty J Dharamsi S Drynan D. International fieldwork placements in low-income countries: exploring community perspectives. Aust Occup Ther J (2016) 63(5):321–8. 10.1111/1440-1630.12291 27111028

[17] CAPTE Accreditation Handbook. PT Standards and Required Elements. (2016). Available from: http://www.capteonline.org/AccreditationHandbook/

[18] Ganzer CA Barnes A Uphold C Jacobs AR Transient ischemic attack and cognitive impairment: a review. J Neurosci Nurs (2016) 48(6):322–7. 10.1097/JNN.0000000000000236 27824800

[19] Williams RS Biel AL Dyson BJ Spaniol J. Age differences in gain- and loss-motivated attention. Brain Cogn (2017) 111:171–81. 10.1016/j.bandc.2016.12.003 28038367

[20] Firlag M Kamaszewski M Gaca K Bałasińska B. Age-related changes in the central nervous system in selected domestic mammals and primates. Postepy Hig Med Dosw (Online) (2013) 67:269–75. 10.5604/17322693.1044490 23619226

[21] Shaffer J. Neuroplasticity and clinical practice: building brain power for health. Front Psychol (2016) 7:1118. 10.3389/fpsyg.2016.01118 27507957 PMC4960264

[22] Shaw CA Lanius RA van den Doel K. The origin of synaptic neuroplasticity: crucial molecules or a dynamical cascade? Brain Res Brain Res Rev (1994) 19(3):241–63. 10.1016/0165-0173(94)90014-0 7820132

[23] Apple DM Solano-Fonseca R Kokovay E Neurogenesis in the aging brain. Biochem Pharmacol (2017) 141:77–85. 10.1016/j.bcp.2017.06.116 28625813

[24] Cai W Zhang K Li P Zhu L Xu J Yang B Dysfunction of the neurovascular unit in ischemic stroke and neurodegenerative diseases: an aging effect. Ageing Res Rev (2017) 34:77–87. 10.1016/j.arr.2016.09.006 27697546 PMC5384332

[25] Alfonso Uresti-Cabrera L Vaca-Palomares I Diaz R Beltran-Parrazal L Fernandez-Ruiz J. Effects of aging on strategic-based visuomotor learning. Brain Res (2015) 1618:9–16. 10.1016/j.brainres.2015.05.022 26014620

[26] Creighton AS Davison TE Kissane DW. The correlates of anxiety among older adults in nursing homes and other residential aged care facilities: a systematic review. Int J Geriatr Psychiatry (2017) 32(2):141–54. 10.1002/gps.4604 27753141

[27] Salter KL Foley NC Jutai JW Teasell RW. Assessment of participation outcomes in randomized controlled trials of stroke rehabilitation interventions. Int J Rehabil Res (2007) 30(4):339–42. 10.1097/MRR.0b013e3282f144b7 17975455

[28] Rizzuto D Melis RJF Angleman S Qiu C Marengoni A. Effect of chronic diseases and multimorbidity on survival and functioning in elderly adults. J Am Geriatr Soc (2017) 65(5):1056–60. 10.1111/jgs.14868 28306158

[29] Elliott TR Pezent GD. Family caregivers of older persons in rehabilitation. NeuroRehabilitation (2008) 23(5):439–46. 18957730 PMC2597573

[30] Berghuis KM De Rond V Zijdewind I Koch G Veldman MP Hortobágyi T Neuronal mechanisms of motor learning are age dependent. Neurobiol Aging (2016) 46:149–59. 10.1016/j.neurobiolaging.2016.06.013 27494184

[31] Kawahira K Shimodozono M Ogata A Tanaka N. Addition of intensive repetition of facilitation exercise to multidisciplinary rehabilitation promotes motor functional recovery of the hemiplegic lower limb. J Rehabil Med (2004) 36(4):159–64. 10.1080/16501970410029753 15370731

[32] Baker MK Atlantis E Fiatarone Singh MA. Multi-modal exercise programs for older adults. Age Ageing (2007) 36(4):375–81. 10.1093/ageing/afm054 17537741

[33] Noohi F Boyden NB Kwak Y Humfleet J Müller ML Bohnen NI Interactive effects of age and multi-gene profile on motor learning and sensorimotor adaptation. Neuropsychologia (2016) 84:222–34. 10.1016/j.neuropsychologia.2016.02.021 26926580 PMC4849282

[34] Berghuis KMM Semmler JG Opie GM Post AK Hortobágyi T. Age-related changes in corticospinal excitability and intracortical inhibition after upper extremity motor learning: a systematic review and meta-analysis. Neurobiol Aging (2017) 55:61–71. 10.1016/j.neurobiolaging.2017.03.024 28431286

[35] Naudé J Paz JT Berry H Delord B. A theory of rate coding control by intrinsic plasticity effects. PLoS Comput Biol (2012) 8(1):e1002349. 10.1371/journal.pcbi.1002349 22275858 PMC3261921

[36] Heuer H Hegele M. Learning new visuo-motor gains at early and late working age. Ergonomics (2007) 50(7):979–1003. 10.1080/00140130701240828 17510818

[37] Scheerer NE Tumber AK Jones JA. Attentional demands modulate sensorimotor learning induced by persistent exposure to changes in auditory feedback. J Neurophysiol (2016) 115(2):826–32. 10.1152/jn.00799.2015 26655821

[38] van Breda E Verwulgen S Saeys W Wuyts K Peeters T Truijen S Vibrotactile feedback as a tool to improve motor learning and sports performance: a systematic review. BMJ Open Sport Exerc Med (2017) 3(1):e000216 10.1136/bmjsem-2016-000216 PMC5530110 28761708

[39] Wishart LR Lee TD. Effects of aging and reduced relative frequency of knowledge of results on learning a motor skill. Percept Mot Skills (1997) 84(3 Pt 1):1107–22. 10.2466/pms.1997.84.3.1107 9172230

[40] Carmeli E Aging, neuroplasticity and neuro rehabilitation. Aging Sci (2014) 2:e110–1. 10.4172/2329-8847.1000e110

[41] Muratori LM Lamberg EM Quinn L Duff SV. Applying principles of motor learning and control to upper extremity rehabilitation. J Hand Ther (2013) 26(2):94–102. 10.1016/j.jht.2012.12.007 23598082 PMC3773509

[42] Sidaway B Ala B Baughman K Glidden J Cowie S Peabody A Contextual interference can facilitate motor learning in older adults and in individuals with Parkinson’s disease. J Mot Behav (2016) 48(6):509–18. 10.1080/00222895.2016.1152221 27340809

[43] Volery S Singh N de Bruin ED List R Jaeggi MM Mattli Baur B Traditional balance and slackline training are associated with task-specific adaptations as assessed with sensorimotor tests. Eur J Sport Sci (2017) 17(7):838–46. 10.1080/17461391.2017.1317833 28488937

[44] Lam LC Chau RC Wong BM Fung AW Tam CW Leung GT A 1-year randomized controlled trial comparing mind body exercise (Tai Chi) with stretching and toning exercise on cognitive function in older Chinese adults at risk of cognitive decline. J Am Med Dir Assoc (2012) 13(6):568.e15–20. 10.1016/j.jamda.2012.03.008 22579072

[45] Shen X Wong-Yu IS Mak MK Effects of exercise on falls, balance, and gait ability in Parkinson’s disease: a meta-analysis. Neurorehabil Neural Repair (2016) 30(6):512–27. 10.1177/1545968315613447 26493731

[46] Vaughan-Graham J Cott C Wright FV The Bobath (NDT) concept in adult neurological rehabilitation: what is the state of the knowledge? A scoping review. Part II: intervention studies perspectives. Disabil Rehabil (2015) 37(21):1909–28. 10.3109/09638288.2014.987880 25427891

[47] Vaughan-Graham J Cott C Wright FV The Bobath (NDT) concept in adult neurological rehabilitation: what is the state of the knowledge? A scoping review. Part I: conceptual perspectives. Disabil Rehabil (2015) 37(20):1793–807. 10.3109/09638288.2014.985802 25411026

[48] Levin MF Panturin E. Sensorimotor integration for functional recovery and the Bobath approach. Motor Control (2011) 15(2):285–301. 10.1123/mcj.15.2.285 21628730

[49] Stock R Thrane G Anke A Gjone R Askim T Early versus late-applied constraint-induced movement therapy: a multisite, randomized controlled trial with a 12-month follow-up. Physiother Res Int (2017). 10.1002/pri.1689 28686338

[50] Ballester BR Maier M San Segundo Mozo RM Castañeda V Duff A Verschure PFMJ. Counteracting learned non-use in chronic stroke patients with reinforcement-induced movement therapy. J Neuroeng Rehabil (2016) 13(1):74. 10.1186/s12984-016-0178-x 27506203 PMC4979116

[51] Sharman MJ Cresswell AG Riek S. Proprioceptive neuromuscular facilitation stretching: mechanisms and clinical implications. Sports Med (2006) 36(11):929–39. 10.2165/00007256-200636110-00002 17052131

[52] Sharma V Kaur J. Effect of core strengthening with pelvic proprioceptive neuromuscular facilitation on trunk, balance, gait, and function in chronic stroke. J Exerc Rehabil (2017) 13(2):200–5. 10.12965/jer.1734892.446 28503533 PMC5412494

[53] Osternig LR Robertson R Troxel R Hansen P. Muscle activation during proprioceptive neuromuscular facilitation (PNF) stretching techniques. Am J Phys Med (1987) 66(5):298–307. 10.1097/00002060-198710000-00009 3434629

[54] Ahlskog JE Geda YE Graff-Radford NR Petersen RC. Physical exercise as a preventive or disease-modifying treatment of dementia and brain aging. Mayo Clin Proc (2011) 86(9):876–84. 10.4065/mcp.2011.0252 21878600 PMC3258000

[55] Clark DJ Christou EA Ring SA Williamson JB Doty L. Enhanced somatosensory feedback reduces prefrontal cortical activity during walking in older adults. J Gerontol A Biol Sci Med Sci (2014) 69(11):1422–8. 10.1093/gerona/glu125 25112494 PMC4229993

